# Intussusception in Incisional Hernia: A Case Report and Literature Review

**DOI:** 10.7759/cureus.49346

**Published:** 2023-11-24

**Authors:** Mohamed Hassan, Shelby V Bryant, Ahmed A Saad, Syed Shah

**Affiliations:** 1 General Surgery, Maidstone and Tunbridge Wells NHS Trust, Kent, GBR; 2 Orthogeriatrics, Maidstone and Tunbridge Wells NHS Trust, Kent, GBR

**Keywords:** surgical management of intussusception, bowel obstruction, incarcerated incisional hernia, incisional hernia, intussusception

## Abstract

Intussusception in adults is a rare condition. Most frequently, intussusception involves the small intestine and, very rarely, the large intestine. In this report, we present the case of a 79-year-old male who was admitted with symptoms and signs of bowel obstruction due to an incarcerated incisional hernia (a tender irreducible incisional hernia associated with nausea and vomiting). His CT scan confirmed intussusception in his incisional hernia, showing the target sign. An emergency laparotomy, small bowel resection, and anastomosis were done. The histopathology report revealed the cause of intussusception to be a polypoid small bowel B cell lymphoma. It is necessary to excise the affected bowel segment in order to treat adult intussusception because it is commonly associated with malignant organic lesions. Computed tomography is the most sensitive imaging modality for intussusception; thus, we must consider a low threshold for a scan for patients presenting with abdominal pain.

## Introduction

A single case of adult intussusception occurs in every 12,000 hospital admissions [1]. In 70% to 90% of cases, an organic lesion occurs within the intussusception. Adult intussusception may indicate malignancy underlying long-lasting, intermittent, and non-specific symptoms, with abdominal pain being the most common presenting symptom [1]. Computed tomography imaging is becoming more frequent in evaluating patients with abdominal pain, resulting in a growing number of preoperative intussusception diagnoses.

## Case presentation

A 79-year-old male presented to the emergency department of our district general hospital experiencing a tender, irreducible incisional hernia associated with nausea and vomiting. He was on the waiting list for an elective hernia repair, but COVID cancellations meant he was delayed. He noted the night before that his hernia had increased in size. His background history included asthma, hypertension, and abdominal prostatectomy.

**Figure 1:**
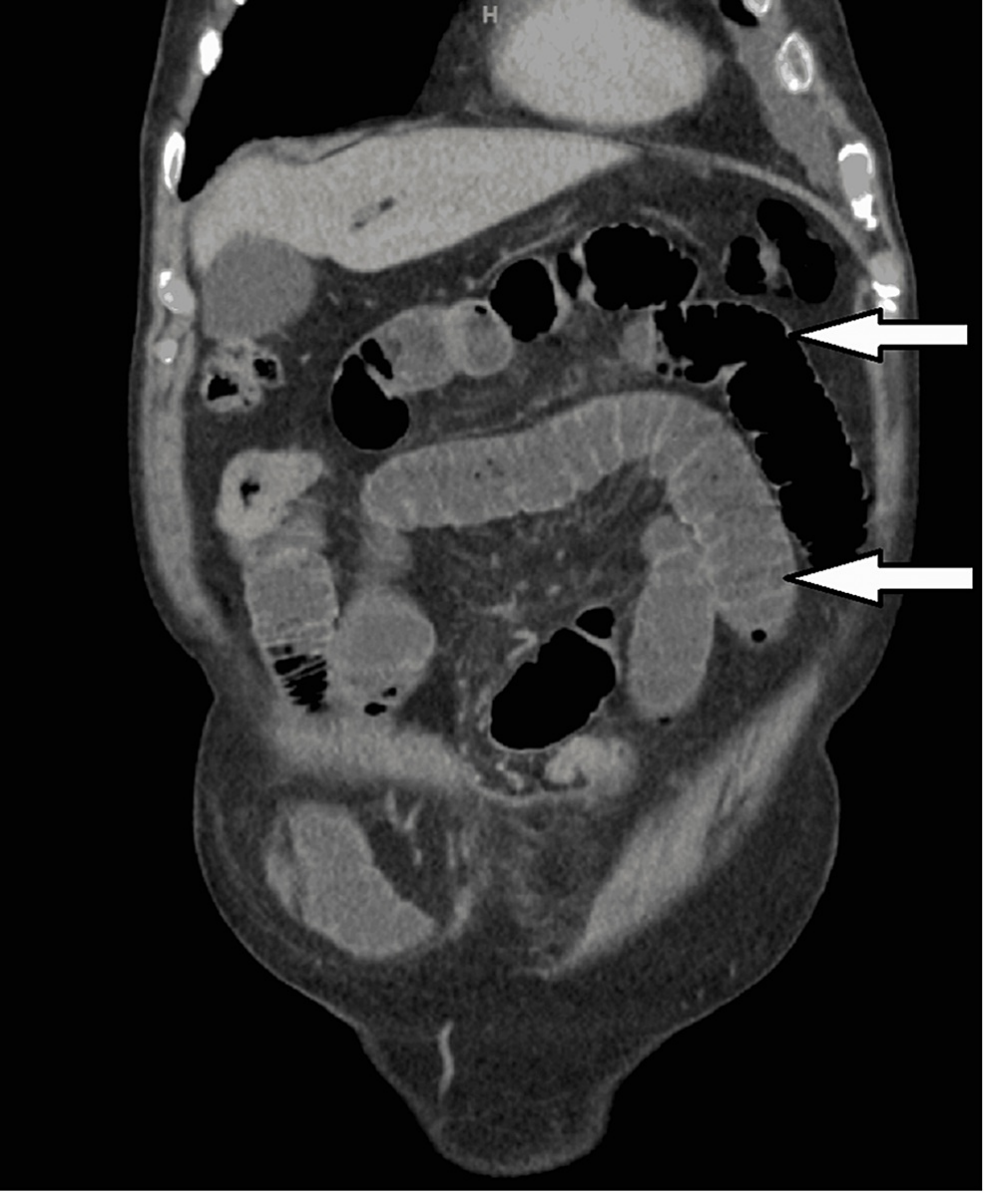
A coronal abdominal CT scan image shows small bowel loop dilatation due to obstruction (white arrows).

**Figure 2:**
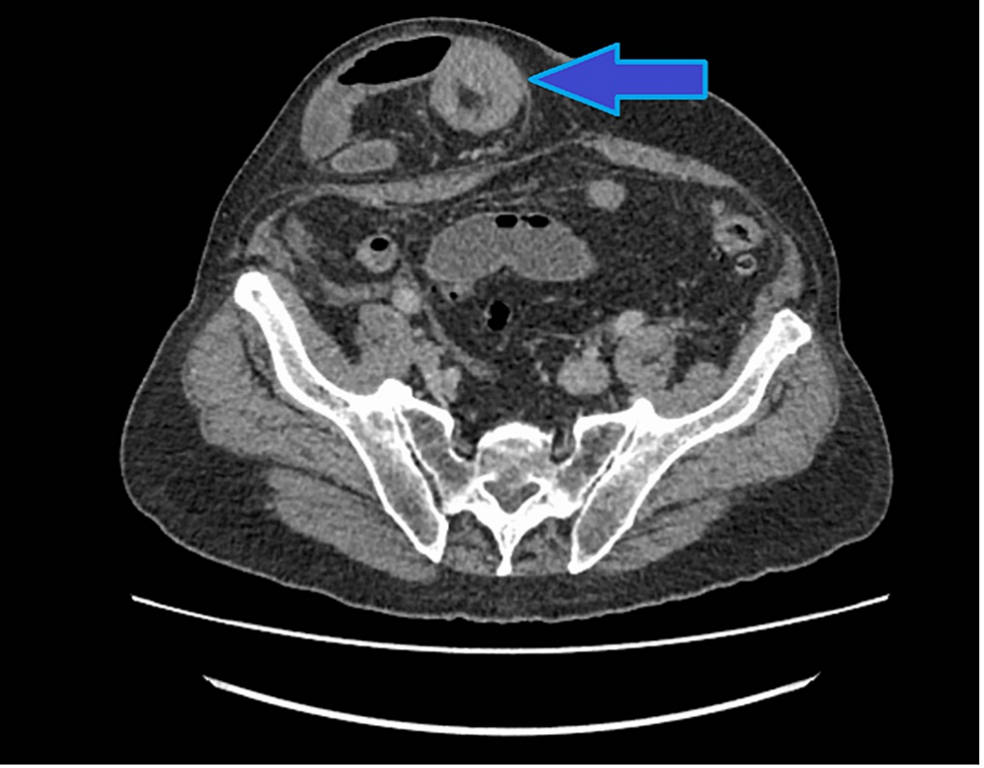
An axial abdominal CT scan image shows intussusception in the incisional hernia and the target sign (blue arrow).

On examination, he had abdominal distension and a tender, irreducible incisional hernia in the right lower quadrant. His inflammatory markers were mildly raised (white blood cells: 9.79 x 109/L, C-reactive protein: 47 mg/L, haemoglobin: 107 g/L). The patient was admitted to the hospital, and a CT scan of the abdomen and pelvis was arranged, which revealed a small bowel obstruction secondary to the incarceration of an incisional hernia (Figures 1-2).

He was offered emergency surgery. A laparotomy confirmed a small bowel intussusception. An incision was made over the site of the hernia, and the decision for laparotomy over laparoscopy was brought about by both the patient's history of previous laparotomies and the consultant's preference. He had a small bowel resection and a primary anastomosis. He recovered well on the ward postoperatively. He was back on a soft diet, and his bowels functioned normally prior to his discharge.

A macroscopic examination of the resected part reported that the specimen consisted of a non-orientated small bowel segment with features of intussusception, measuring in total about 80 mm in length and 60 mm in diameter. No perforation sites were seen. The cut surface at the intussusception point revealed a pale-coloured, ill-defined polypoidal lesion, with areas of haemorrhage encompassing the prolapsing portion.

**Figure 3:**
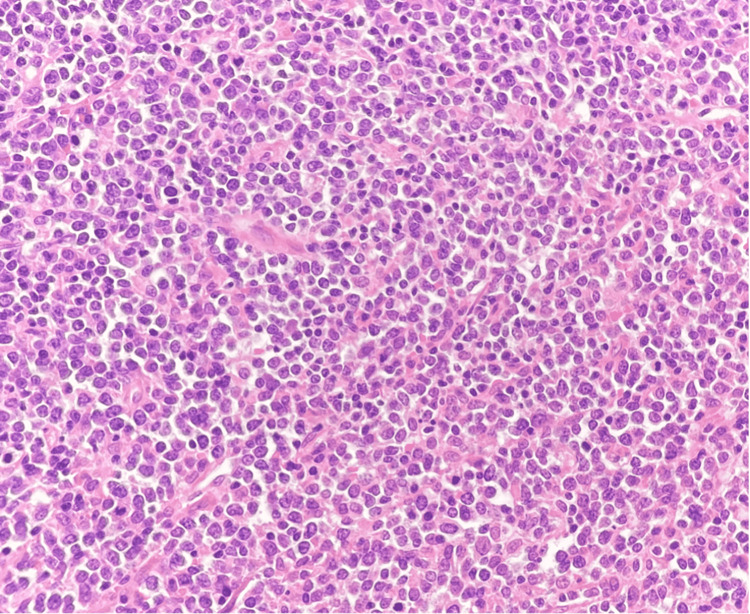
A high-power H&E-stained section shows large atypical lymphoid cells with irregular nuclei, coarse chromatin, and nucleoli.

**Figure 4:**
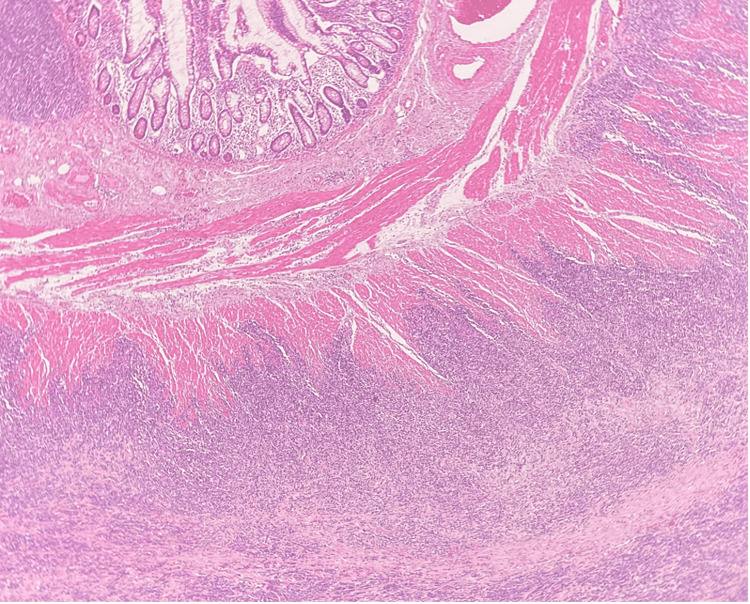
A low-power H&E-stained section shows infiltration of tumour cells through the muscularis propria of the small bowel.

Microscopic examination sections from the polypoid lesion showed an atypical infiltrate of intermediate to large lymphoid cells with irregular nuclei, coarse chromatin, and small nucleoli (Figure 3), with infiltration of tumour cells through the muscularis propria of the small bowel (Figure 4).

On immunohistochemistry, the atypical lymphoid infiltrate showed positivity with CD45, CD20, CD79a, CD10, and Bcl-6. The features are those of a high-grade B cell lymphoma, most in keeping with the diffuse large B cell lymphoma germinal centre phenotype.

## Discussion

Intussusception is a difficult diagnosis that warrants a high degree of clinical suspicion in adults. The challenges arise because abdominal pain is not only one of the most common complaints evaluated in the emergency department but also generally non-specific in nature. The severity of signs and symptoms present during a physical evaluation plays a crucial role in how abdominal pain is assessed and managed. It can be hard to diagnose intussusception in adults because it mimics many alternative diagnoses [2]. If misdiagnosed, it can result in severe complications that can negatively impact the patient.

It represents 1% of small bowel obstruction in adults. Nearly 90% of cases are linked to an underlying pathological process, most commonly a neoplasm [1-3].

Most frequently, intussusception involves the small intestine and, very rarely, the large intestine. The most common symptoms are cramping abdominal pain, nausea, bloating, and bloody stools [4-5]. A CT of the abdomen is the most sensitive diagnostic method for making a preoperative diagnosis of adult intussusception, especially when patients present with non-specific abdominal pain. Ultrasound abdominal imaging has a lower sensitivity for detecting adult intussusception than abdominal CT, but it can identify the characteristic target sign in some cases, especially in patients who have palpable abdominal masses [6].

Intussusception in adults often requires surgical intervention due to the high incidence of malignancy [7]. Most intussusceptions occur at the junctions of segments that move freely and those that are retroperitoneally or adherently fixed [8]. There are four categories of intussusceptions based on their location: entero-enteric, involving only the small intestine; colo-colic, affecting only the large bowel; ileo-colic, caused by the prolapse of the terminal ileum in the ascending colon; and ileocecal, where intussusception takes place at the ileocecal valve. In patients with ileocolic, ileocecal, and colo-colic intussusceptions, resection with appropriate oncologic techniques is recommended due to the high incidence of bowel malignancy underlying these conditions [9].

In cases of gastroduodenal and coloanal intussusceptions, surgical treatment is sometimes difficult, and innovative approaches may be necessary. It is feasible to perform minimally invasive surgery, especially when the diagnosis is made with confidence prior to surgery.

Wani presented a similar case in June 2009, showing jejunal intussusception in incisional hernia secondary to adhesions [10]. That was the only other case in the literature presenting intussusception in an incisional hernia.

## Conclusions

We present a rare case of intussusception in an incisional hernia. This illustrates the importance of considering the possibility of uncommon presentations and outcomes. Surgeons face a difficult challenge when dealing with intussusception in adults. Due to non-specific symptoms that are often subacute, preoperative diagnosis is usually missed or delayed. A CT scan of the abdomen is considered the most sensitive imaging modality for diagnosing intussusception and determining whether it has a lead point or not. It is necessary to excise the affected bowel segment in order to treat adult intussusception because it is commonly associated with malignant organic lesions. If the segment involved is viable or if malignancy is not suspected, reduction can be attempted in small bowel intussusceptions.
